# Modeling the Regional Distribution of International Travelers in Spain to Estimate Imported Cases of Dengue and Malaria: Statistical Inference and Validation Study

**DOI:** 10.2196/51191

**Published:** 2024-05-27

**Authors:** David García-García, Beatriz Fernández-Martínez, Frederic Bartumeus, Diana Gómez-Barroso

**Affiliations:** 1 Department of Communicable Diseases, National Centre of Epidemiology Instituto de Salud Carlos III Madrid Spain; 2 Epidemiology and Public Health Biomedical Network Research Consortium (CIBERESP) Madrid Spain; 3 Group of Theoretical and Computational Ecology, Centre for Advanced Studies of Blanes Spanish Research Council Blanes Spain; 4 Ecological and Forestry Applications Research Centre Barcelona Spain; 5 Catalan Institution for Research and Advanced Studies Barcelona Spain

**Keywords:** epidemiology, imported infections, modeling, surveillance system, vector-borne diseases

## Abstract

**Background:**

Understanding the patterns of disease importation through international travel is paramount for effective public health interventions and global disease surveillance. While global airline network data have been used to assist in outbreak prevention and effective preparedness, accurately estimating how these imported cases disseminate locally in receiving countries remains a challenge.

**Objective:**

This study aimed to describe and understand the regional distribution of imported cases of dengue and malaria upon arrival in Spain via air travel.

**Methods:**

We have proposed a method to describe the regional distribution of imported cases of dengue and malaria based on the computation of the “travelers’ index” from readily available socioeconomic data. We combined indicators representing the main drivers for international travel, including tourism, economy, and visits to friends and relatives, to measure the relative appeal of each region in the importing country for travelers. We validated the resulting estimates by comparing them with the reported cases of malaria and dengue in Spain from 2015 to 2019. We also assessed which motivation provided more accurate estimates for imported cases of both diseases.

**Results:**

The estimates provided by the best fitted model showed high correlation with notified cases of malaria (0.94) and dengue (0.87), with economic motivation being the most relevant for imported cases of malaria and visits to friends and relatives being the most relevant for imported cases of dengue.

**Conclusions:**

Factual descriptions of the local movement of international travelers may substantially enhance the design of cost-effective prevention policies and control strategies, and essentially contribute to decision-support systems. Our approach contributes in this direction by providing a reliable estimate of the number of imported cases of nonendemic diseases, which could be generalized to other applications. Realistic risk assessments will be obtained by combining this regional predictor with the observed local distribution of vectors.

## Introduction

Throughout history, human mobility has been a key determinant for the spread of infectious diseases. From the 14th century bubonic plague pandemic to the 1918 Spanish flu as well as the more recent Ebola epidemic and COVID-19 pandemic, the way individuals travel across the globe has shaped the evolution and geographical dynamics of infectious diseases [1-3].

International mobility flows are especially relevant for the spread of vector-borne diseases (VBDs), which often receive less attention in routine epidemiological surveillance plans in countries where they are not endemic. In recent years, global warming and intensified urbanization processes have favored the establishment of previously foreign species around the globe, such as *Aedes, Anopheles*, and *Culex* mosquitoes [4-7]. These are vectors for malaria, dengue, yellow fever, West Nile virus, Zika, and chikungunya, and thus, they pose a significant public health risk that needs adequate preparedness [8-10]. Air travel plays a central role in the diffusion of most of these diseases, allowing their spread through imported cases at nonendemic locations [11-13] with vector presence, and it must be incorporated in decision-support systems to achieve operational preparedness and risk prediction [14-16]. Reliable descriptions and predictions of migration flows have been proven to be valuable tools for the design of more effective public health policies [17-20]. However, new developments are always needed as complex dynamics are expected to arise from the multi-step life cycle of VBDs [21-23].

A wide range of approaches have been used to model risks of imported cases of dengue [24-27] and malaria [28-30] from endemic to nonendemic regions. Several studies [31-33] have incorporated data on the global airline network to assist outbreak prevention and public health preparedness. However, accurate estimations of how such imported cases disseminate locally after arrival in the receiving countries are harder to devise. While fine-grained data on local mobility are available from cellular networks [34-36], it is not clear how the specific behavior of travelers can be differentiated from the local population dynamics. Moreover, travel-related data are usually obtained through coarse-grained spatial statistics, thus involving large territories. A reason for this is the inherent complexity and range of scales involved in human flows. While it is possible to record information at designated locations (eg, airports), it is much more difficult to reliably collect detailed movement data of target groups over larger geographical regions. In these cases, insights on the nature of human traveling behaviors and motivations that uncover hidden patterns in these processes are crucial as they may be used to sidestep the need for excessively detailed and thus unreachable data [37-39].

We aimed to provide accurate descriptions of how infected travelers may distribute in a territory, which could be valuable input for local authorities in the design of cost-effective VBD prevention and control strategies. For this, we approximated the local distribution of travelers arriving at a specific country (or any other territory) in terms of readily available indicators, rather than considering travel information that is usually not quantified at a local scale. These statistics gauge the appeal of each region to foreign travelers, quantifying the number of imported cases each region may receive. We calibrated our model with the number of imported cases of dengue and malaria at each province in Spain from 2015 to 2018 and then performed validation by comparing our model’s estimates for the number of imported cases in 2019 with official data.

## Methods

### Travelers’ Distribution

**Figure 1:**
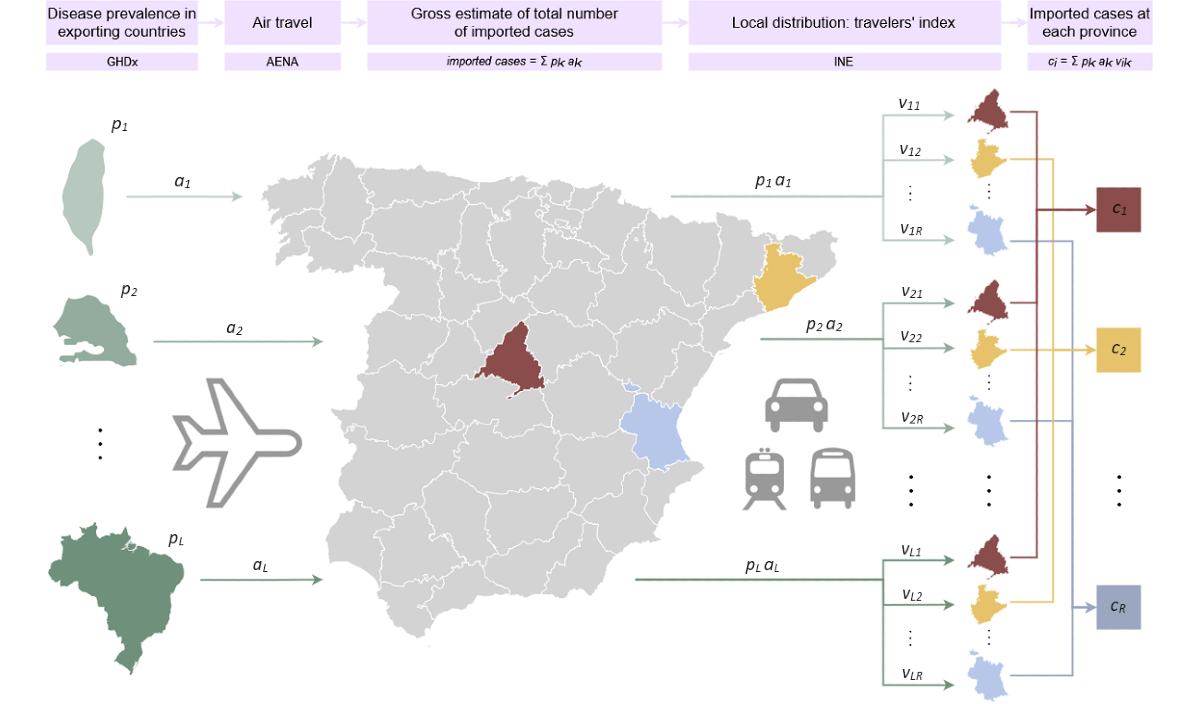
Summary of the rationale behind our approach. Infected travelers arrive at the importing country from *L* exporting countries across the world by means of air travel. For each country *k*, the prevalence of the disease in the country (*p_k_*) and the number of travelers arriving from it to the importing country (*a_k_*) are combined to obtain an estimate of the number of imported cases (*p_k_ a_k_*). These spread across *R* regions or spatial units of the importing country following a distribution that can be estimated by means of the “travelers’ index.” The index is computed from local statistics concerning economic and touristic activity and the number of foreign residents at each region. The travelers' index *v_ki_* measures the proportion of imported cases from country *k* moving to local region *i* upon arrival at the importing country. The total number of cases expected to arrive at the region (*c_i_*) is then obtained as the sum of the estimated number of imported cases over all exporting countries: *c_i_*=∑*p_k_ a_k_* *v_ki_*. AENA: Aeropuertos Españoles y Navegación Aérea (Spanish Airports and Aerial Navigation); GHDx: Global Health Data Exchange; INE: Instituto Nacional de Estadistica (Spanish National Statistics Institute).

We first developed a theoretical framework to estimate how travelers distribute throughout the territory after their arrival in the country. See Figure 1 for a schematic description of this approach.

### Input Data

We used the following yearly statistics from 2015 to 2019 to compute the relative appeal of each province in Spain to international travelers, which are publicly available and curated by the Spanish National Statistics Institute [40]:

- Tourist indicators: For both hotels and tourist apartments, we used the variables total capacity, number of national travelers, number of foreign travelers, number of overnight stays by national travelers, and number of overnight stays by foreign travelers.
- Economic indicators: We considered each province’s population, gross domestic product (GDP), GDP per capita, number of private limited companies (Sociedades Limitadas), and number of public limited companies (Sociedades Anónimas).
- Indicators for visits to friends and relatives: For each country and province, we used the number of foreign residents by nationality, number of foreign residents by birthplace, and number of national residents by birthplace (other than Spain).

The following 3 additional data inputs were used in our approach:

- Arrival data: We computed the yearly number of travelers arriving in Spain from 2015 to 2019 at each of the 100 airports with the largest flows of incoming travelers and aggregated publicly available monthly data provided by the public entity in charge of the Spanish Airports and Aerial Navigation (Aeropuertos Españoles y Navegación Aérea, AENA) [41].
- Disease data: We used yearly prevalence estimates for malaria and dengue from 2015 to 2019 provided by Global Health Data Exchange (GHDx) [42]. Data on malaria were supplied by the Malaria Atlas Project [43].
- Cases in Spain: The number of imported cases of malaria and dengue (including any type of infection by the dengue virus) is reported by each province to the Spanish National Surveillance System (Red Nacional de Vigilancia Epidemiológica, RENAVE). These data were used.

### Travelers’ Index

We have described the rationale used to combine the above statistics in an informed indicator to estimate the propensity of international travelers to move to a specific region. We considered a country that receives travelers from other countries of the world (these are denoted as *importing* and *exporting* countries, respectively). We assumed that the importing country is divided into *regions*, which may represent geographical regions or administrative units, for instance.

Motivations for international travel are usually classified into 3 major categories: tourism, business, and visits to friends and relatives [44,45]. Based on this principle, we computed the relative importance of each region in the importing country in terms of each of these drivers as follows: *t_i_* measures the relative importance of region *i* in terms of tourism, *e_i_* measures the relative importance of region *i* in terms of economy, and *r_ik_* measures the relative importance of region *i* in terms of visits to friends and relatives for travelers arriving from country *k*.

The last indicator depends on the exporting country *k*. For instance, when computing the travelers’ index with *k*=Brazil, only the number of Brazilian residents is considered in the computation. The 3 indicators were computed from several available statistics concerning the regions of the country (see the Input Data subsection) as the relative contribution of each region to the country’s total. For instance, when using GDP to measure economic status, the relative importance of region *i* in terms of economy would be as follows:

*t_i_* = GDP of region *i* / total GDP of the importing country **(1)**

We then computed the travelers’ index for each region *i* of the importing country and any exporting country *k* as the average of the 3 indicators as follows:

*v_ik_* = 1/3 (*t_i_* + *e_i_* + *r_ik_*) **(2)**

It follows from equations 1 and 2 that for each exporting country *k*, the sum of the travelers’ index of the country over all the regions *i* of the importing country is as follows:

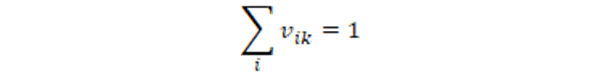



Therefore, given an exporting country *k*, the travelers’ index *v_ik_* may be understood as an estimate of the portion of travelers *a_k_* arriving from this country to each province *i*. Hence, we can estimate the total number of travelers arriving at a given region *i* as follows:

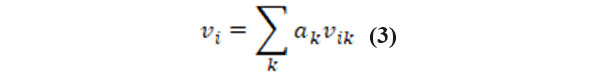



where the sum runs over all exporting countries *k*, and *a_k_* is the total number of travelers arriving from any such country *k*. Accordingly, we estimated the number of imported cases of a disease at each province *i* as the sum of the imported cases from each of the exporting countries *k* as follows:

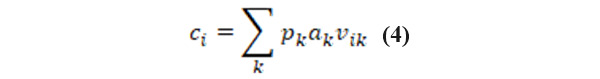



where *p_k_* denotes the prevalence of the disease in the exporting country *k*. Plainly speaking, we estimated the total number of cases arriving from country *k* as the product of the total number of travelers arriving from the country and the prevalence of the disease in the country. We then estimated how these cases disseminate across regions by means of the travelers’ index, which assigns to travelers a relative importance or preference for each of the regions. Adding these local distributions over all the exporting countries resulted in the total number of expected cases at each of the regions in the importing country. See Figure 1 for a visual representation of this reasoning.

### Model Calibration and Validation

**Figure 2:**
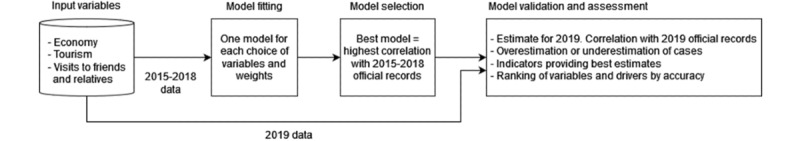
Summary of the model building process. Key steps include the fitting, selection, validation, and assessment of the model.

In order to test the validity of our approach, we followed the pipeline depicted in Figure 2. The steps involved in this process were (1) input variables, (2) model fitting, (3) model selection, and (4) model validation and assessment.

#### Input Variables

We considered data from Spain (importing country) and its 52 provinces (regions over which the imported cases disseminate). We computed the relative importance of *t_i_*, *e_i_*, and *r_ik_* using each of the statistics listed in the Input Data subsection for each of the 3 drivers (ie, tourism, economy, and visits to friends and family).

#### Model Fitting

We used equation 2 to construct the travelers’ index (1 for each possible combination of the indicators). We combined these with arrivals and prevalence data to obtain estimates for the expected number of cases at each province for 2015-2018 (equation 4), resulting from simple averages of the indicators *t_i_*, *e_i_*, and *r_ik_*. We also considered a generalized version of the travelers’ index by replacing the average in equation 2 with a weighted average as follows:

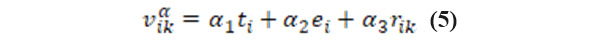



where 0 ≤ *a_j_* ≤ 1 and *a_1_* + *a_2_* + *a_3_* = 1. The coefficient *a_j_* measures the relative importance given to each of the 3 categories themselves (tourism, business, and visits to friends and relatives) in a particular model. For instance, an estimate obtained from a value of *a_1_* close to 1 and values of *a_2_* and *a_3_* close to 0 in equation 5 assumes that tourism is the most important driver for travelers from countries where the disease is endemic than business or visits to friends and relatives. Identifying the choice of the weight *a_j_* and indicator in equation 5 that provide a better estimate allows us to also understand which of the 3 drivers among *t_i_*, *e_i_*, and *r_ik_* plays a more significant role in the motivation of travelers carrying the disease.

#### Model Selection

To identify which travelers’ index best approximates the number of imported cases per province, we computed the correlation between the estimates provided by each of our models (1 for each combination of indicators) and the actually reported cases of malaria and dengue at each province for the years 2015 to 2018. The model reporting the highest value for this correlation was selected as the best model. We followed the same procedure for the case of the weighted averages, and a different estimate was obtained for each choice of the indicator and weight *a_j_*.

#### Model Validation and Assessment

As a final test for accuracy, we computed the correlation between the best model’s estimates for 2019 (data not used during the fitting and selection process) and the officially reported cases for this year, both for the simple and weighted averages. In case this correlation was high, we considered the model as validated and proceeded to the next step.

We assessed 3 features of the resulting model. First, we fit a linear model explaining the estimated number of imported cases at each province in 2019 in terms of the officially reported cases. The coefficient of the linear model may be understood as the number of cases predicted by the model per officially reported case, thus informing of the overestimation or underestimation of the model’s prediction of actually reported cases. This refers only to the raw number of cases as the accuracy of the distribution is captured by the validated correlation. Second, we ranked the contribution of each of the statistics considered in the model by computing the average correlation with 2019 official data of those estimates obtained from models including each particular variable. We also computed the average loss of accuracy associated with each variable as the difference in the average correlation of those models including and not including each statistic. This allowed us to identify which statistics among the choices made for each indicator *t_i_*, *e_i_*, and *r_ik_* provided more reliable predictors of the disease. Third, we followed an analogous procedure for the assessment of the weighted averages and computed the average correlation of those models built from each choice of the indicator and weight *a_j_*. Those indicators scoring higher for larger values of the corresponding weight were expected to inform about the motivations of the travelers carrying each disease among business, tourism, and visits to friends and relatives.

### Human Mobility Model

Finally, we tested the validity of our results against a well-established model for human mobility [46,47], assuming that the movement of travelers does not follow motivations different from those of resident populations. For this, we assumed that, on arrival to their destination airport in Spain, a proportion *q* of imported cases stay at that destination and the rest move to a different province following a well-known, generic, and random human mobility pattern [47,48]. The probability of these travelers moving to each province in Spain is assumed to follow a decaying power law with exponent *γ* on the distance *d* between the origin and destination province centroids (*p*(*d*) = *d*^−^*^γ^*).

We then grouped the total number of expected cases at each province as the sum of those arriving at the province according to their final flight destination and those arriving from any other province by means of other transport modes reflected in a geographically bounded power law distribution. This model was then fitted to the data on the officially reported cases of dengue and malaria from 2015 to 2018 for values of *q* between 0 and 1 (proportion of travelers who leave their destination province upon arrival) and values of *γ* between 1 and 5 (exponent of the power law, with lower values favoring longer-ranged movement and higher values favoring shorter-ranged movement).

We followed an analogous philosophy for the model building and assessment process as in the travelers’ index model. The parameters leading to the highest correlation with the reported cases for the period of 2015 to 2018 were used to compute an estimate for 2019, and the correlation between this estimate and the 2019 official record was then computed to allow for comparison with the travelers’ index model. A linear model between the human mobility model’s estimate and the official 2019 record was also fitted to assess the underestimation or overestimation of the model.

### Ethical Considerations

Our study used publicly available aggregated secondary data with no characteristics that allowed for individual identification. There are no relevant data protection and privacy issues to report.

## Results

### Input Data

A preliminary analysis showed that among all statistics used, those concerning the same drivers were usually highly co-dependent, with some exceptions (eg, GDP per capita; see Figures S1 and S2 in Multimedia Appendix 1 [40]). The relative importance appearing in the computation of the travelers’ index (equation 2) showed little variation over the years (see Figure S3 in Multimedia Appendix 1). This temporal stability has been observed before in the distribution of international [37] and national [49] human mobility flows across destinations, which have been obtained from expressions analogous to equation 1.

The 100 airports with the highest number of incoming travelers were located in 49 countries and accounted for 99.75% of the total incoming travelers to Spain from 2015 to 2019. Out of these countries, 10 were removed from our study as neither malaria nor dengue was present during the time span under study (according to prevalence data from GHDx), resulting in 39 exporting countries. Table 1 shows the number of incoming travelers from each of these countries and the average prevalences of dengue and malaria from 2015 to 2019 as provided by GHDx.

**Table 1:** Exporting countries.

Country^a^	Incoming travelers^b^	Malaria prevalence (/100,000 population)^b^	Dengue prevalence (/100,000 population)^b^
United States	6,845,337	0	0.54
Brazil	3,472,397	78.96	65.40
Colombia	3,053,635	197.47	54.97
Argentina	2,972,990	0	15.47
Peru	2,101,286	323.49	41.37
Mexico	2,063,577	7.26	37.29
Dominican Republic	1,611,336	1.50	49.11
Algeria	1,562,772	4.92	0
Venezuela	1,241,154	1065.28	48.17
Cuba	1,062,531	0	37.93
Cape Verde	1,054,106	135.17	36.41
Ecuador	1,027,244	86.97	37.84
Costa Rica	810,214	0	67.74
Senegal	630,192	2464.71	32.43
Panama	566,765	115.70	56.47
Bolivia	556,092	181.43	60.84
Gambia	522,809	4237.62	33.78
Egypt	466,705	0	10.88
Thailand	365,318	68.10	58.10
Singapore	360,619	0	68.44
Equatorial Guinea	360,393	32981.86	39.18
China	359,372	0.14	24.63
Pakistan	306,321	536.27	41.90
Mauritania	300,521	4095.70	24.57
El Salvador	293,976	8.95	130.85
Republic of Korea	254,457	15.80	0
Nigeria	200,813	18792.47	38.73
Jordan	182,431	0	12.45
Angola	176,690	11182.79	27.97
Guatemala	174,030	169.12	45.25
Saudi Arabia	166,730	3.98	15.52
Ghana	149,374	18512.96	40.65
Guinea	78,545	30131.12	38.35
The Bahamas	49,010	0	39.30
Gabon	38,016	15756.90	44.25
Jamaica	14,802	0	45.43
South Africa	12,394	36.43	0
Cameroon	8994	19904.66	34.96
Mali	1165	16024.21	31.19

^a^Countries with no malaria or dengue prevalence have been removed from the list.

^b^Total incoming travelers and average malaria and dengue prevalences (total cases per 100,000 inhabitants) from 2015 to 2019 for the 39 exporting countries considered in our study, ranked by the number of travelers.

### Estimates and Model Assessment

High correlation values were found for both malaria (0.94) and dengue (0.86) between the best model’s estimates for 2019 and the notified cases. The models that provided the most accurate estimates included public limited companies, foreign travelers at hotels, and foreign residents by birthplace in the computation of the travelers’ index. The same variables led to the best estimates for both malaria and dengue. While considering weighted averages in the construction of the travelers’ index did not improve the accuracy of the models, different motivations were obtained for travelers carrying each of the diseases: economy seemed to best capture the appeal of each region for imported cases of malaria (relative weight of 0.7, with GDP being the most accurate indicator) and visits to friends and relatives seemed to be the main motivation for travelers with dengue (relative weight of 0.9, assigned to the number of foreign residents in the province by birthplace). Different proportions of overestimation were found for each disease (99% for malaria and 86.5% for dengue). A summary of the relevant features of the models provided by the fitting and selection process is presented in Table 2.

**Table 2:** Summary of the models that most accurately approximated the reported cases in 2015-2018.

Disease (model)	Economic indicator (weight^a^)	Tourist indicator (weight^a^)	Visits to friends and relatives indicator (weight^a^)	Pearson correlation of model’s estimate with 2019 data	Overestimation
Malaria^b^ (simple)	Public limited companies	Foreign travelers at hotels	Foreign residents by birthplace	0.94	98.9%
Malaria^b^ (weighted)	GDP^c^ (0.7)	Foreign travelers at hotels (0.1)	Foreign residents by birthplace (0.2)	0.94	99.0%
Dengue^b^ (simple)	Public limited companies	Foreign travelers at hotels	Foreign residents by birthplace	0.86	86.5%
Dengue^b^ (weighted)	No contribution (0)	Foreign travelers at hotels (0.1)	Foreign residents by birthplace (0.9)	0.87	86.7%

^a^For the models including weighted averages, the weight *a_i_* of each indicator is included in parenthesis. If the weight of a given indicator is 0, no contribution to the estimate is provided by the corresponding indicator.

^b^Each row shows the statistics that provide the best estimate of imported cases of each disease, the correlation with the actually reported data in 2019, and the approximation for the proportion of overestimation as obtained from the linear models.

^c^GDP: gross domestic product.

**Figure 3:**
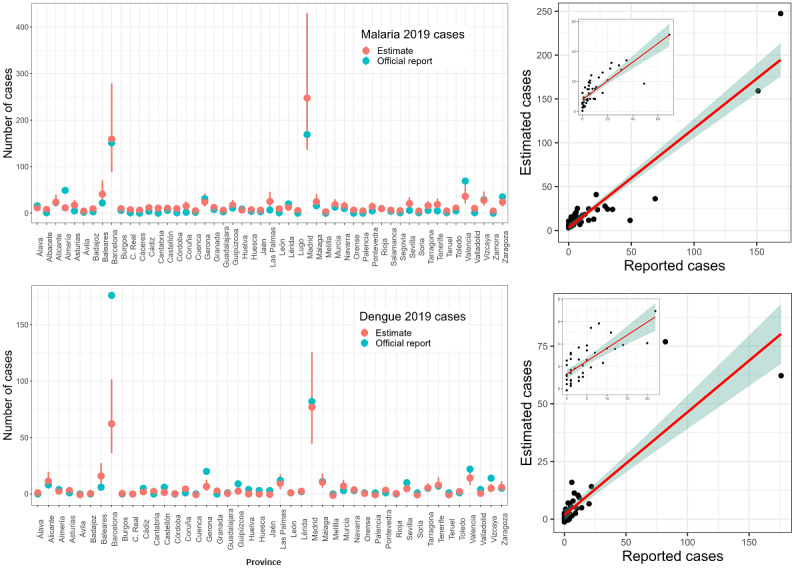
Summary of the best linear models for 2019 imported cases of malaria (top row) and dengue (bottom row). The left column shows the predictions of the models (in red), together with the number of reported cases (in blue) for 2019 at each province in Spain. The right column shows the fit between the estimates of the models and the official records (inset figures correspond to the fit after removing Madrid and Barcelona from the data set).

Figure 3 shows the fit of the weighted models and their estimates for 2019, together with the officially reported number of cases of each disease (malaria and dengue) at each province in Spain. Upon visual inspection, 2 provinces seemed to have a high influence on the fit of the models. These corresponded to Madrid and Barcelona, which hosted a much larger number of reported cases of both diseases. We excluded these provinces from the input data set and repeated the analysis (Table S1 and Figure S4 in Multimedia Appendix 1). While a decrease in correlation was found overall (approximately 0.12 over all models), the resulting estimates still showed high agreement with the official report for 2019 (above 0.74 correlation with 2019 data; see Figure 3, and Table S1 and Figure S4 in Multimedia Appendix 1).

We performed a residual analysis to check for normality and autocorrelation of the residuals of the models. The malaria model showed close-to-normal residuals with no autocorrelation (statistically significant W=0.67 and DW=2.03 in the Shapiro-Wilk and Durbin-Watson tests, respectively). For the dengue model, a relevant deviation was caused by the estimate for Barcelona (Figure 3). Exclusion of this outlier resulted in normally distributed and not autocorrelated residuals (statistically significant W=0.94 and DW=1.87). See Table S3 in Multimedia Appendix 1 for complete details on the residual analysis.

### Variable Performance

The models constructed using simple averages provided a unanimous choice of indicators associated with tourism, economy, and visits to friends and relatives. On the contrary, the best weighted models included different economic indicators. GDP provided the best estimate for imported malaria cases, while no influence of the economic indicator was considered in the best dengue model. In addition, different drivers were the most important ones for each disease, as shown by the much higher relative weight for economic motivations in malaria cases and for visits to friends and relatives in dengue cases (Table 2).

When ranking the contribution of each of the variables to the accuracy of the models, similar results were found for both diseases, with some minor variations across variables (Table 3). Several statistics concerning tourism ranked the highest in this classification, although several others ranked in a low position, indicating that appropriate choices of indicators may be important and may need careful examination. All economic indicators provided an improvement (or absence of a decrease) in correlation, except for GDP per capita, which resulted in less accurate estimates (average decrease of approximately 0.08 in correlation with 2019 data). Indicators corresponding to visits to friends and relatives had mild average effects on the outputs of the models (the largest variation in correlation with 2019 data was −0.02).

**Table 3:** Contribution of each variable to model accuracy.

Variable	Malaria^a^	Malaria (loss)^b^	Dengue^a^	Dengue (loss)^b^
National travelers in hotels	0.83	0.08	0.74	0.05
Overnight stays by national travelers in hotels	0.83	0.07	0.73	0.05
Foreign travelers in hotels	0.82	0.07	0.76	0.08
Total hotel capacity	0.81	0.05	0.74	0.05
Public limited companies (Sociedades Anónimas)	0.81	0.05	0.72	0.04
National travelers in tourist apartments	0.79	0.03	0.70	0.01
Overnight stays by national travelers in tourist apartments	0.79	0.03	0.70	0.02
GDP^c^	0.78	0.02	0.71	0.02
Private limited companies (Sociedades Limitadas)	0.78	0.02	0.70	0.01
Foreign residents by country of nationality	0.77	0.01	0.70	0.01
Foreign residents by country of birth	0.77	0.01	0.70	0.01
Population	0.76	0.00	0.69	0.00
Overnight stays by foreign travelers in hotels	0.75	−0.01	0.69	0.00
National residents by country of birth	0.75	−0.02	0.68	−0.02
Total tourist apartment capacity	0.71	−0.06	0.64	−0.06
GDP per capita	0.69	−0.09	0.63	−0.07
Foreign travelers in tourist apartments	0.67	−0.10	0.62	−0.08
Overnight stays by foreign travelers in tourist apartments	0.63	−0.15	0.58	−0.13

^a^The average correlation of the estimates of the models including each variable in their fit with the officially reported 2019 data.

^b^The average difference in correlation between models including each variable in their fit and models not including each of the variables (variables ranked by the average correlation for predictions).

^c^GDP: gross domestic product.

**Figure 4:**
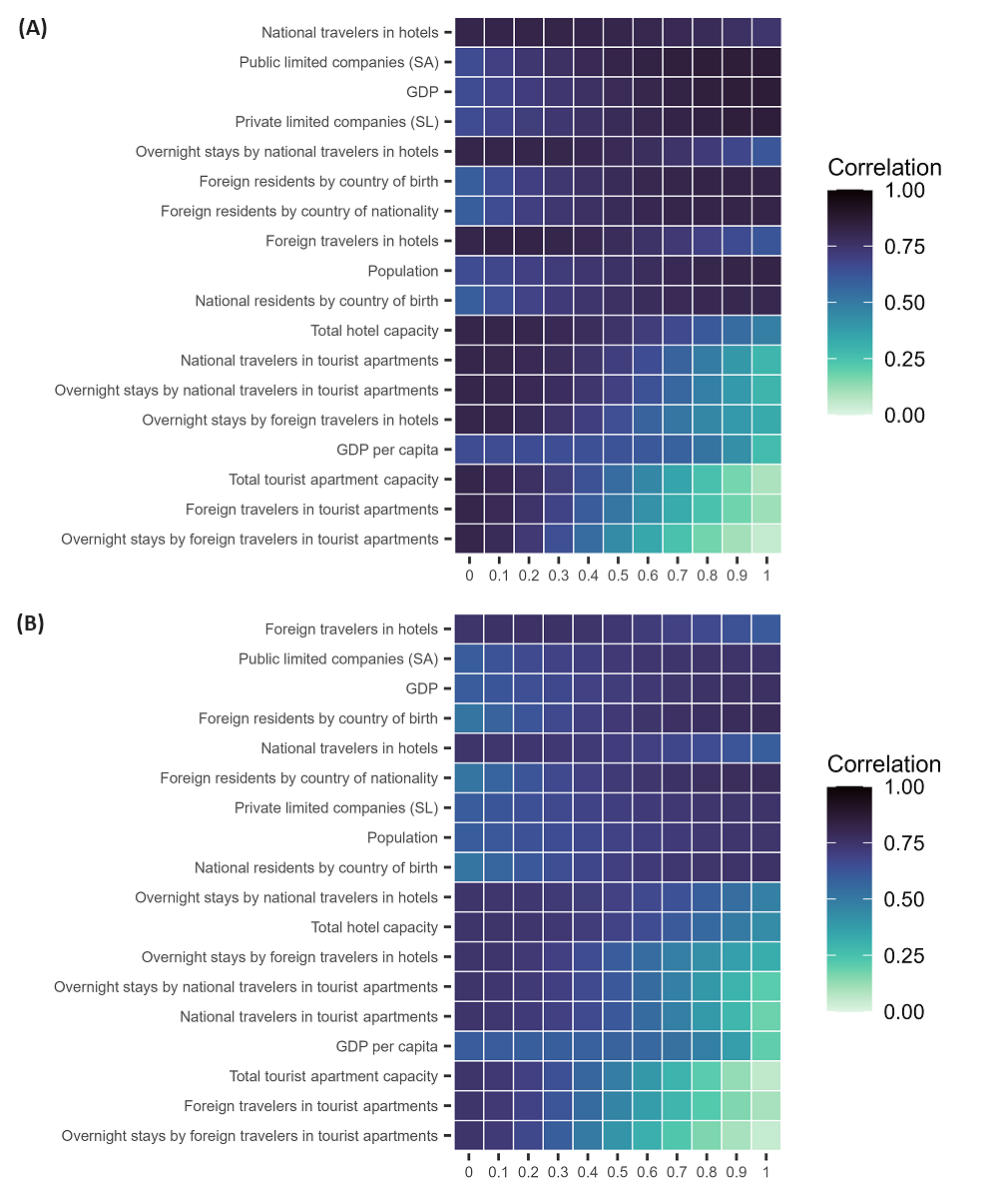
Summary of each input variable’s performance on the estimates for malaria (A) and dengue (B). Each square in the figure is colored according to the average correlation between the official 2019 reports and the estimates provided by the weighted models including each of the variables, with the associated weight ranging from 0 (no contribution from the variable is assumed in the model) to 1 (the model only includes that variable). The variables are ranked from top to bottom according to the overall average correlation with 2019 data of the estimates of the models including each variable. GDP: gross domestic product; SA: Sociedades Anónimas; SL: Sociedades Limitadas.

A similar procedure was followed for the weighted models. The average correlation between the models including each variable and the 2019 official data was computed in this case with stratification by the weight assigned to the variable (Figure 4). In addition to the ranking of variables (similar to data in Table 3), this provided a measure of the variability of each variable’s contribution to the accuracy of the model in terms of the weight assigned to it. Smaller overall variations in model accuracy were identified for variables measuring visits to friends and relatives, for instance, while much larger variability was recorded for some tourist indicators. This shows the higher potential loss in accuracy that would result from including these variables in the models than including other variables.

### Comparison With a Generic Mobility Model

For both dengue and malaria, the human mobility models ranked higher in terms of correlation with 2019 data for higher values of the assumed proportion of travelers who do not move from their destination province upon arrival (*q*; see Figures S5 and S6 in Multimedia Appendix 1). Models also favored the choice of smaller values of the exponent of the power law distribution (highest average correlation with 2019 data for *γ*=1), indicating that longer movements may take place if a displacement occurs after arrival. Much higher variability in the correlation with 2019 data was due to the choice of *q* than the choice of *γ* (Figure S6 in Multimedia Appendix 1).

In general, the estimates of the generic mobility model for the distribution of imported cases were less accurate when compared to actually reported cases than those resulting from the travelers’ index models. This was the case for both malaria and dengue (0.59 and 0.66 correlation with 2019 data, respectively; Table 4). In both cases, the best estimate was obtained assuming that imported cases were indeed reported at the region of arrival via air travel in Spain (*q*=1), signaling that international travelers more often choose their final destination as the end of their trip and rendering the choice of *γ* (the scaling exponent associated with the length of the displacement) arbitrary. Visual examination of the resulting fit revealed a high influence of some provinces in the results, as in the travelers’ index (Madrid, Barcelona, and Las Palmas for malaria; Madrid and Barcelona for dengue). We performed the same analysis after removing these provinces from the input data set and identified a strong decrease in the correlation of the model estimates for the number of imported cases with the reported cases for 2019 (0.003 and 0.12 for malaria and dengue, respectively). See Table S2 and Figures S5 and S6 in Multimedia Appendix 1 for more detailed information on the generic human mobility model.

**Table 4:** Summary of the human mobility model that most accurately approximated the reported cases from 2015 to 2018 (including all provinces).

Model	Proportion of cases that do not move after arrival (*q*)	Exponent of the power law distribution (*γ*)	Correlation with 2019 data	Overestimation
Malaria^a^	1	Any	0.59	99.5%
Dengue^a^	1	Any	0.66	95.2%

^a^Each row shows the parameters of the model that provide the best estimate of imported cases of each disease, the correlation with the actually reported data in 2019, and the overestimation of the models as obtained from the linear fit with official records.

## Discussion

We computed estimates for the number of imported cases of malaria and dengue at each province in Spain based on simple methodological assumptions. Our approach makes use of readily available data and provides approximations of the actually declared number of cases of the disease. This advance may contribute to the adequate modeling and monitoring of VBDs, which might be relevant for effective outbreak prevention strategies. More efficient resource allocation strategies for both vector control and disease prevention can be designed if reliable predictions of the geographical locations of imported cases are available. By circumventing the need for detailed large-scale data on human mobility or traveler behavior, this methodology is accessible and suitable to be used in countries lacking more exhaustive data infrastructure, for instance [39,50]. The reasoning presented here could also be generalized to other choices of territories.

The high correlation found between our estimates and real data support the validity of our approach based on a priori theoretical conceptualization. This agreement in trend suggests that our estimates are reliable enough for the elaboration of scale-less risk indicators, for instance. On the other hand, our estimates of the raw number of imported cases were simplistic (product of yearly prevalence and total number of travelers), which resulted in substantial overestimation of the number of imported cases. For the case of malaria, this is coherent with the epidemiology of the disease, being more severe unless treatment is available and having a higher incidence in economically deprived populations [51]. These factors may prevent individuals with malaria from engaging in international displacements. For dengue, however, the identified overestimation (8 predicted cases per notified case) lies relatively close to previously obtained estimates of the underreporting of cases in other contexts [52]. This suggests that our approach could also provide a valid method for assessing the sensibility of epidemiological systems. In any case, our focus was on assessing the validity of the travelers’ index as a method to improve risk analysis, rather than developing a predictive model for imported cases of the diseases.

The proposed computation of the key indicators involved in our model (the travelers’ index *v_ik_*) has the advantage of being partially robust considering errors in declaration or incomplete data collection. Indeed, as these only involve the relative importance of each region in the country, correction factors are unnecessary in our approach, and incomplete data will yield equally valid estimates as long as the underreporting can be assumed to be comparable for all regions. Moreover, the little variation in time shown by these quantities (see Figure S3 in Multimedia Appendix 1) could allow for reliable estimates even when only past statistics are available.

A key finding in this direction is that while the impact of each particular indicator in the quality of the estimate was similar for both diseases, the relevant drivers for case importation were different (economic motivations for malaria cases and visits to friends and relatives for dengue cases). This may be due to the different nature of the motivation for international travel across countries in the world. Most malaria cases were imported from African countries, while travelers carrying dengue usually arrived from America or Asia (see Table S1 in Multimedia Appendix 1). Travelers arriving from these continents are expected to follow different motivations for international displacement. Actually, malaria cases imported to Spain in the prepandemic era were mainly due to visits to friends and relatives or migration in almost 75% of cases, corresponding to travelers following economic motivations [53]. On the other hand, dengue cases were imported mainly by tourist travelers or visits to friends and relatives [54].

Further evidence of the appropriateness of our approach was provided by a comparison with the human mobility model. While the validity of this model has been established in many contexts and is widely acknowledged [47], it provided much less accurate estimates and was highly sensitive to data retrieval from provinces with a larger number of disease cases. This demonstrates the need to consider specific designs that take into account travelers’ behaviors and differentiate them from general resident population dynamics.

Future developments of our approach should cover the following improvements:

- Coupling with postimportation dynamics: Our framework could be integrated into more complicated models incorporating transmission dynamics that involve the life cycle of the disease within the vector and the host [55-58]. Well-developed approaches, such as compartmental models, could benefit from more precise estimates on the expected location of arrival of imported cases of diseases.
- Refining the gross estimate of imported cases: As mentioned above, we computed simple estimates for the total number of cases arriving to the importing country (product of yearly prevalence and total number of travelers). We focused on how these cases distribute over the regions of the importing country. Consideration of more elaborate estimates of these quantities or the local distribution of the disease in the exporting countries would probably yield more precise final estimates.
- Extending the scope of the model to other diseases: A particular feature of the treated examples is that virtually all incoming streams of travelers into Spain from regions where malaria and dengue are endemic, which may result in transmission, may be assumed to be associated with air travel. This may not be the case for other diseases and countries, for which detailed data on the total traveler flow or further development of the proposed methodology could be necessary. Similarly, other importation phenomena that may depend on human behavior or allocation of resources could be analyzed under our assumptions, such as passive mobility of vectors by human means of transportation [59] or migratory flows [60,61].

It should be noted that our model was focused on countries with high dengue and malaria prevalences, and hence, they were likely to export these diseases to Spain. However, this concept could be generalized to other types of risk-related importation scenarios like the transport of new vectors or exotic species (invasion biology), which is another crucial process in the spread of VBDs.

Several factors may be limiting the extent of our results. First, both malaria and dengue are diseases known to be subject to high underreporting [51,52]. Second, we validated our models with annual data, as data on the number of monthly reported cases were too noisy. In any case, model predictions could be generated at a higher temporal resolution by incorporating monthly numbers of arriving travelers in the country, for instance. Third, our model was designed to address the motivations of international travelers; however, a significant number of imported cases may correspond to national travelers returning to the country or individuals from other nonendemic regions, especially for dengue. It would be desirable to devise an accurate method to differentiate between these 2 types of travelers and incorporate both motivations in the model. Finally, geographical borders are not always the best spatial human structure [62], and therefore, the availability of data with a finer geographical resolution could result in significant improvements in our estimates. We also note that our model has been validated with data obtained prior to the COVID-19 pandemic, and differences may arise in the postpandemic era. Therefore, further validation with future data is desirable.

We have shown the validity of the travelers’ index as a method to estimate the distribution of imported cases of malaria and dengue from endemic regions. This is an appropriate way to improve disease risk prediction on the basis of human mobility patterns. Our methodology adds value to available socioeconomic information relevant to public health. Nonetheless, human mobility is just 1 component of VBD risk models. The other key components that need to be added are vector (mosquito) distribution and suitability. Our work will be combined with multi-sourced presence/absence and suitability vector data in Spain, including both authoritative and citizen science data collections [63], and integrated into the Spanish National Surveillance System for VBDs. Pairing the risk of importation of cases and the risk of local transmission through the presence of vectors will provide a more comprehensive evaluation of the threats posed by VBDs to public health.

## Appendix

Multimedia Appendix 1 Supporting data and detailed results for the statistical analysis.

## Abbreviations

GDP: gross domestic product
GHDx: Global Health Data Exchange
VBD: vector-borne disease

## Data Availability

Indicators for tourism, economy, and visits to friends and relatives are available from the Spanish National Institute for Statistics [40]. Arrival data are available from the Spanish Aviation and Airports Office [41]. Disease prevalence data for exporting countries are available from Global Health Data Exchange [42]. Reported cases of dengue and malaria in Spain are collected by the National Surveillance System and managed by the Spanish National Center for Epidemiology, and the data are available upon reasonable request (contact email: vigilancia.cne@isciii.es).
