# Supplementary material for: Modeling the Regional Distribution of International Travelers in Spain to Estimate Imported Cases of Dengue and Malaria: Statistical Inference and Validation Study
Source: JMIR Public Health Surveill. 2024 May 27;10:e51191. doi: 10.2196/51191 (PMC11165286; doi:10.2196/51191)
Supplement: Multimedia Appendix 1 [file publichealth_v10i1e51191_app1.pdf]

Multimedia Appendix 1. Supplementary data.

Local indicators

We found substantial correlations between most of the economic and touristic indicators used in the construction of the travelers' index (Figure S1). Among the economic variables, GDP per capita displayed a much lower correlation with the remaining statistics, that shared very high pair-to-pair correlations. The variables quantifying the importance of tourism in the regions were also highly correlated, with those corresponding to hotels showing a higher variability, both among each other and with respect to those corresponding to apartments. Variables across the two different categories showed much smaller correlations in general, with the exception of the high correlations found between the economic variables (other than the GDP per capita) and some of the indicators measuring hotel activity.

	Population	Public ltd comp	Private ltd comp	GDP	GDP per capita	Apartments: national travellers	Apartments: foreign travellers	Apartments: national overnights	Apartments: foreign overnights	Apartments: capacity	Hotels: national travellers	Hotels: foreign travellers	Hotels: national overnights	Hotels: foreign overnights	Hotels: capacity
Population	1.00	0.93	0.99	0.98	0.24	0.42	0.19	0.41	0.13	0.19	0.92	0.70	0.79	0.38	0.58
Public ltd comp	0.93	1.00	0.96	0.96	0.37	0.27	0.09	0.24	0.04	0.06	0.87	0.61	0.67	0.30	0.49
Private ltd comp	0.99	0.96	1.00	0.99	0.30	0.40	0.18	0.39	0.12	0.18	0.93	0.69	0.78	0.37	0.57
GDP	0.98	0.96	0.99	1.00	0.37	0.35	0.14	0.33	0.08	0.12	0.91	0.68	0.74	0.35	0.54
GDP per capita	0.24	0.37	0.30	0.37	1.00	-0.02	-0.05	-0.02	-0.08	-0.10	0.26	0.23	0.13	0.10	0.11
Apartments: national travellers	0.42	0.27	0.40	0.35	-0.02	1.00	0.79	0.96	0.76	0.88	0.56	0.59	0.79	0.65	0.74
Apartments: foreign travellers	0.19	0.09	0.18	0.14	-0.05	0.79	1.00	0.78	0.99	0.93	0.23	0.66	0.49	0.87	0.82
Apartments: national overnights	0.41	0.24	0.39	0.33	-0.02	0.96	0.78	1.00	0.76	0.91	0.50	0.59	0.77	0.66	0.73
Apartments: foreign overnights	0.13	0.04	0.12	0.08	-0.08	0.76	0.99	0.76	1.00	0.93	0.16	0.58	0.43	0.82	0.76
Apartments: capacity	0.19	0.06	0.18	0.12	-0.10	0.88	0.93	0.91	0.93	1.00	0.27	0.54	0.58	0.73	0.73
Hotels: national travellers	0.92	0.87	0.93	0.91	0.26	0.56	0.23	0.50	0.16	0.27	1.00	0.65	0.90	0.37	0.60
Hotels: foreign travellers	0.70	0.61	0.69	0.68	0.23	0.59	0.66	0.59	0.58	0.54	0.65	1.00	0.71	0.90	0.94
Hotels: national overnights	0.79	0.67	0.78	0.74	0.13	0.79	0.49	0.77	0.43	0.58	0.90	0.71	1.00	0.56	0.76
Hotels: foreign overnights	0.38	0.30	0.37	0.35	0.10	0.65	0.87	0.66	0.82	0.73	0.37	0.90	0.56	1.00	0.95
Hotels: capacity	0.58	0.49	0.57	0.54	0.11	0.74	0.82	0.73	0.76	0.73	0.60	0.94	0.76	0.95	1.00

Figure S1. Pair-to-pair correlations between local touristic and economic indicators obtained from INE [40].

The numbers of foreign residents by birthplace showed a very high correlation with those by nationality, as expected (Figure S2). A significantly smaller, nevertheless large correlation was found for nationalized individuals born abroad.

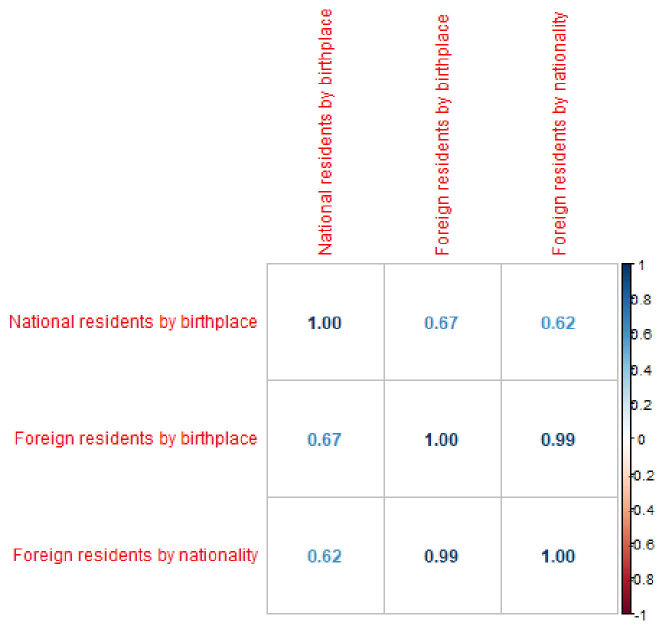

Figure S2. Pair-to-pair correlations between indicators concerning foreign and born-abroad population obtained from INE [40].

As stated in the main text, we found relatively small variations on the relative importances t_i, e_i, r_{ik} over the years for most provinces and choice of statistics (Figure S3). This suggests that the role of the provinces within the national context may be more stable than the global evolution displayed by the country as a whole, and that these indicators may provide reliable projections in the short-term.

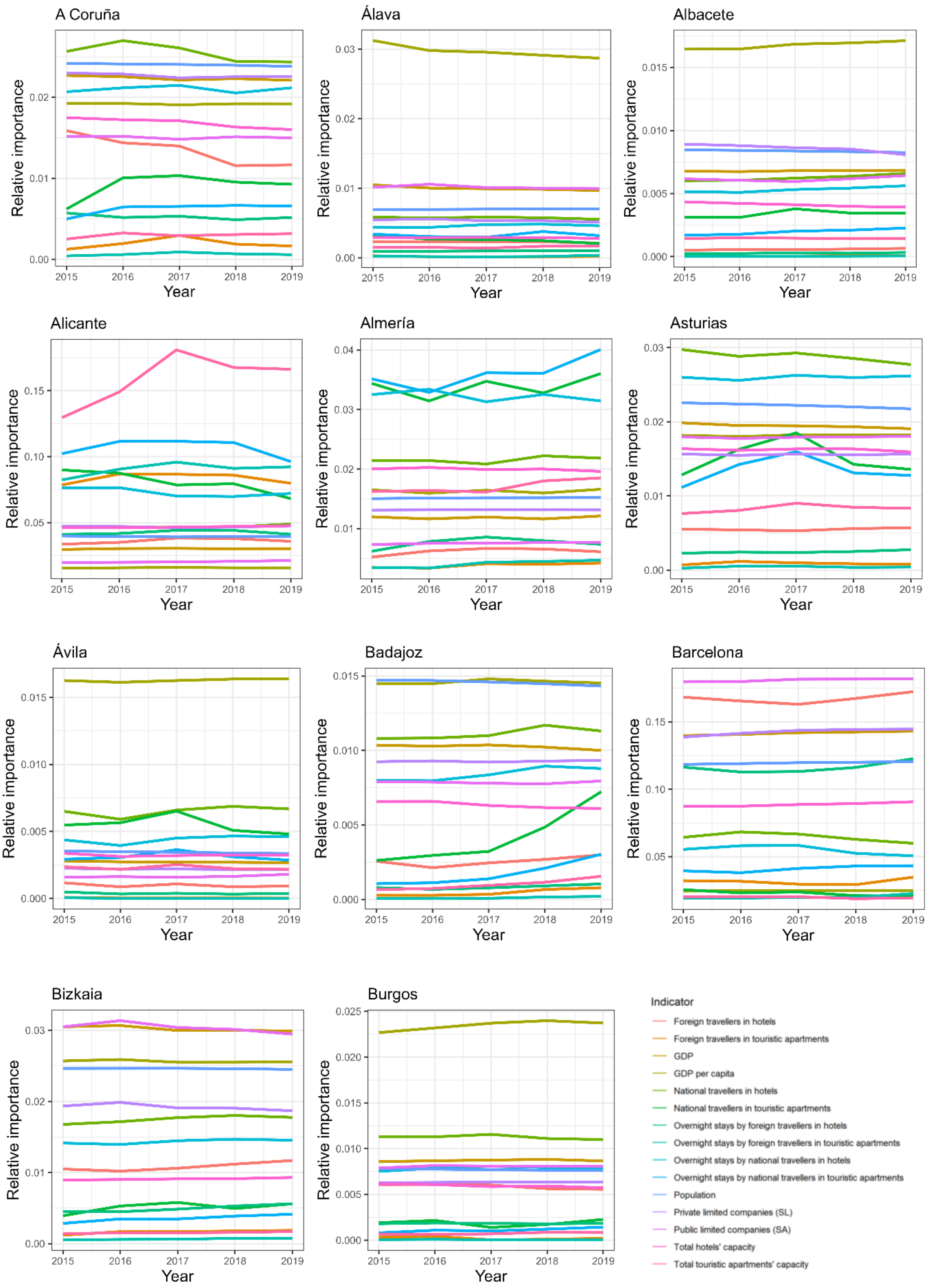

Figure S3.1: Relative importances of provinces in Spain in terms of economic and touristic indicators. Evolution of the indicators e_i and t_i during the years 2015-2019 for several provinces in Spain (see equation (2) in main text) for several choices of statistics.

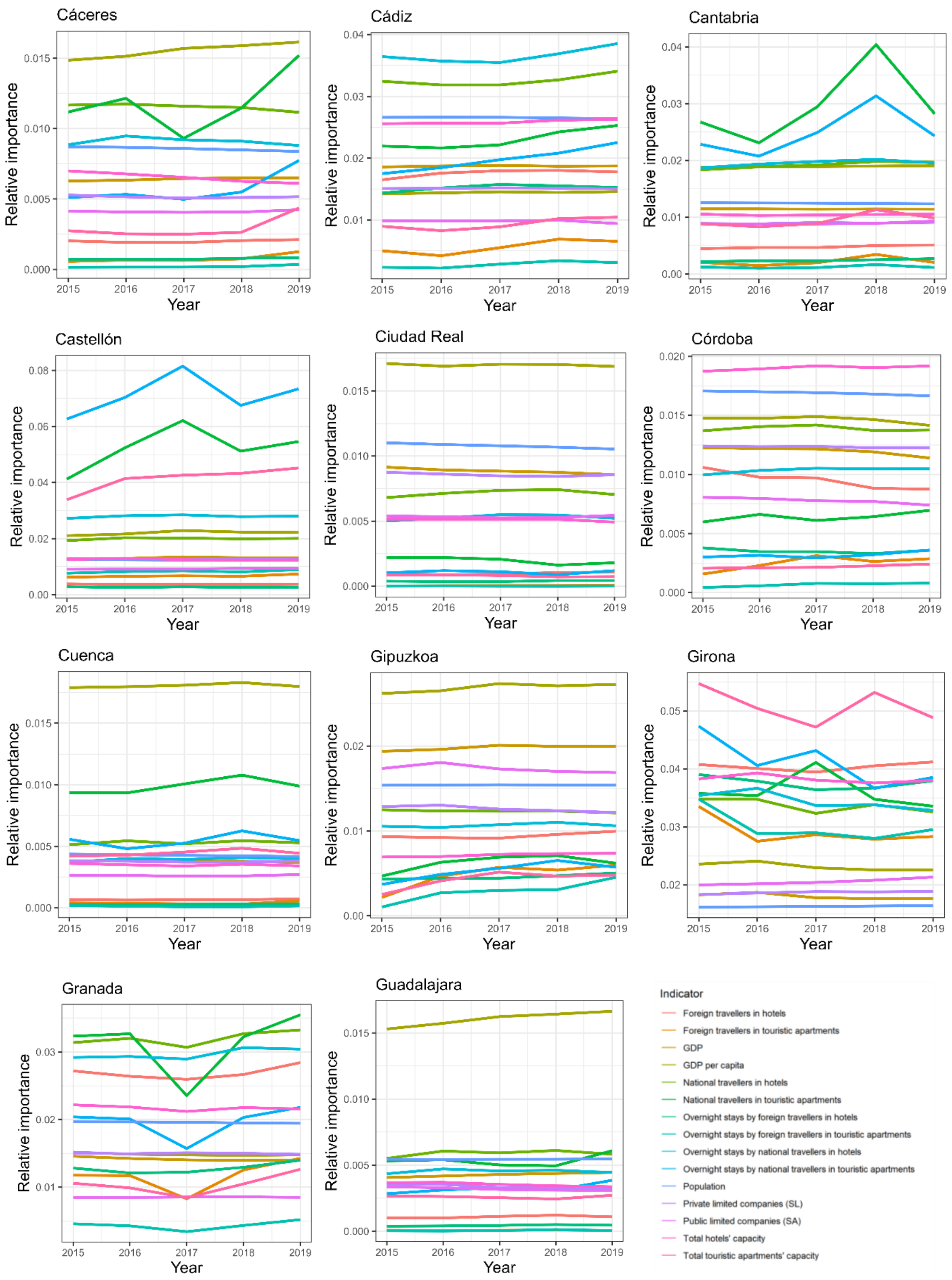

Figure S3.2: Relative importances of provinces in Spain in terms of economic and touristic indicators. Evolution of the indicators e_i and t_i during the years 2015-2019 for several provinces in Spain (see equation (2) in main text) for several choices of statistics.

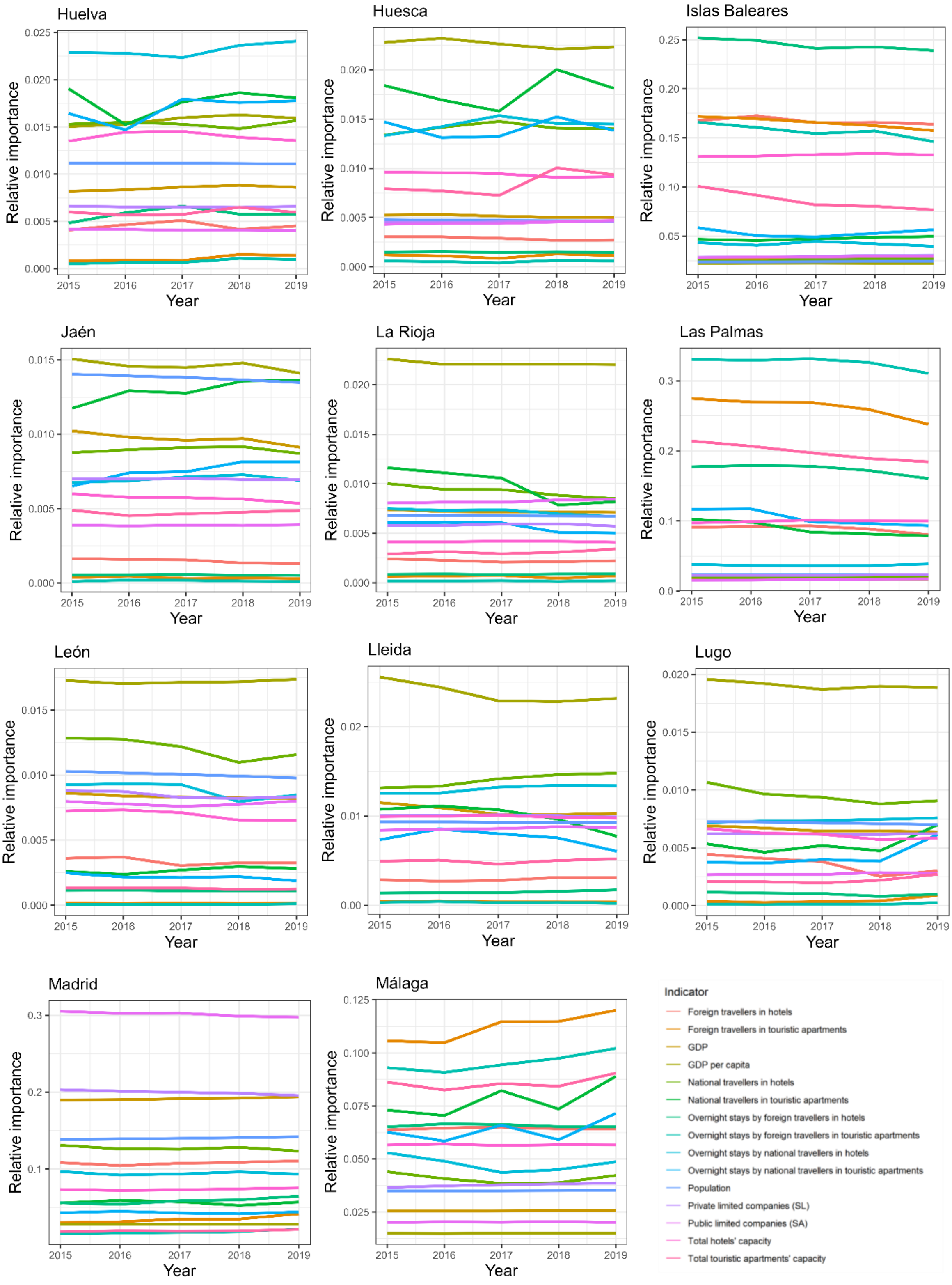

Figure S3.3: Relative importances of provinces in Spain in terms of economic and touristic indicators. Evolution of the indicators e_i and t_i during the years 2015-2019 for several provinces in Spain (see equation (2) in main text) for several choices of statistics.

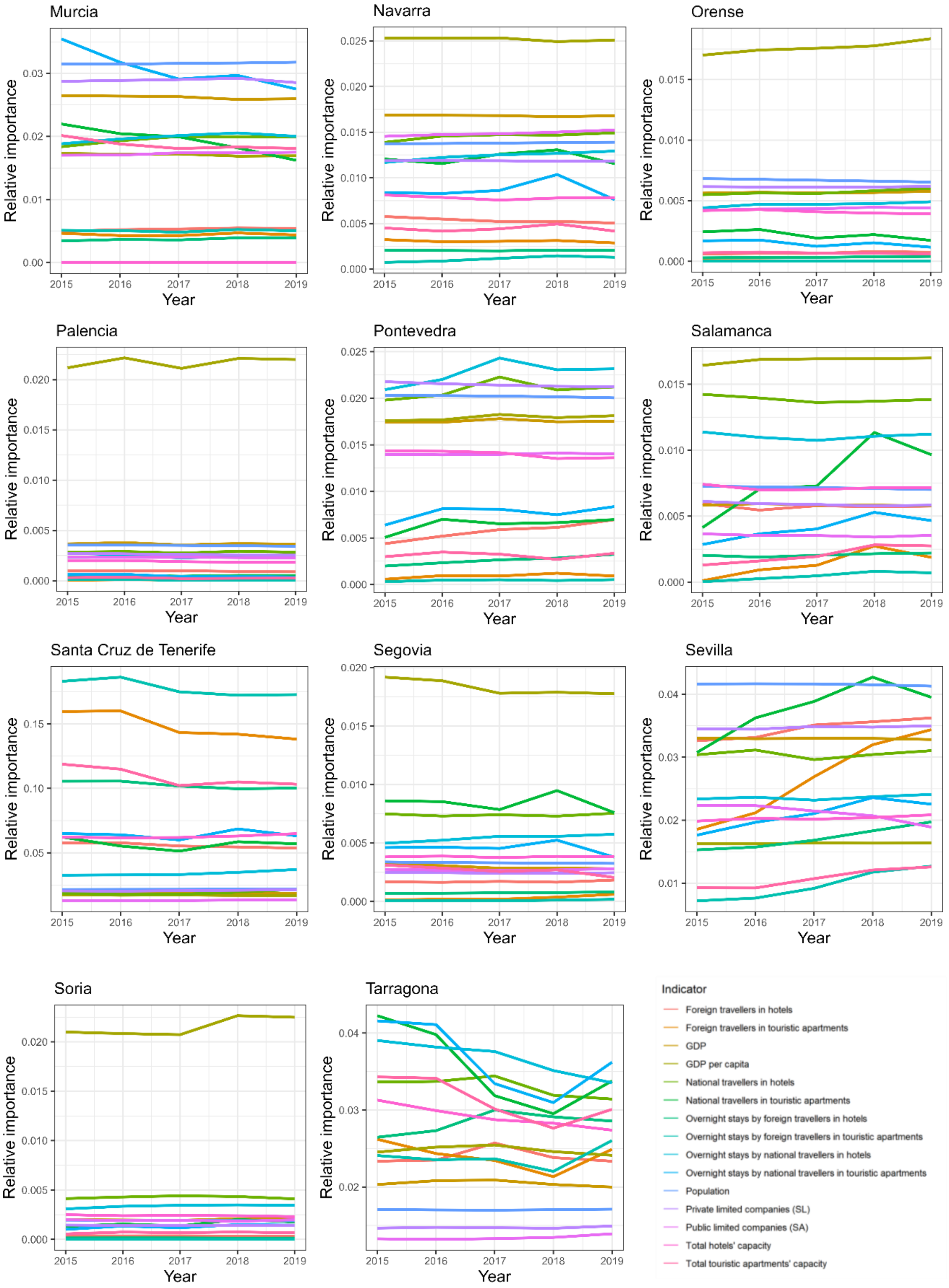

Figure S3.4: Relative importances of provinces in Spain in terms of economic and touristic indicators. Evolution of the indicators e_i and t_i during the years 2015-2019 for several provinces in Spain (see equation (2) in main text) for several choices of statistics.

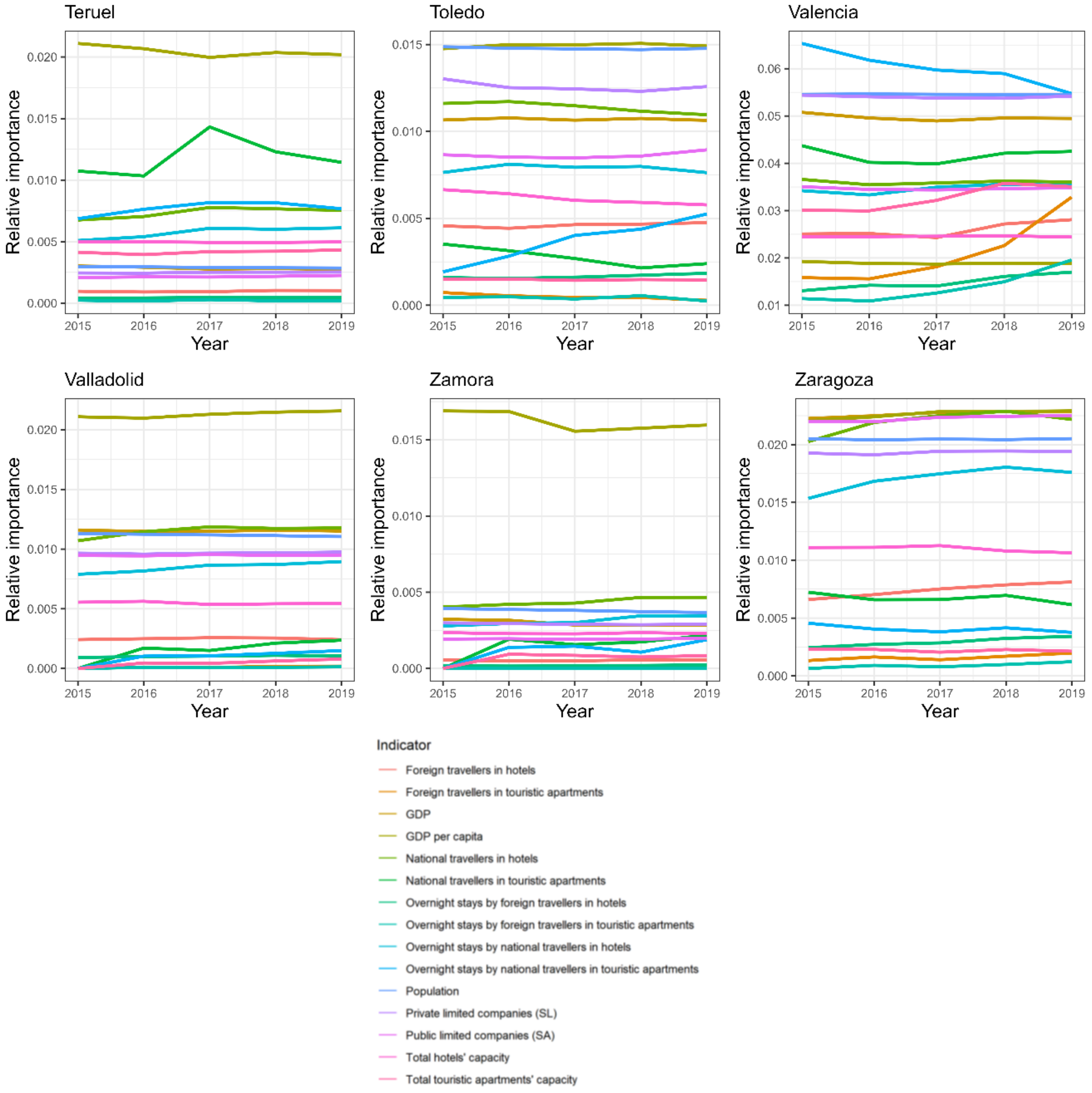

Figure S3.5: Relative importances of provinces in Spain in terms of economic and touristic indicators. Evolution of the indicators e_i and t_i during the years 2015-2019 for several provinces in Spain (see equation (2) in main text) for several choices of statistics.

Excluding outliers from the analysis

Table S1 shows the relevant features of the best models after excluding Madrid and Barcelona from the analysis. This was done in order to test the robustness of our approach, checking to what degree the accuracy of our models was due only to the extreme values of these provinces, both in terms of reported cases and travelers' index (see Figure S3).

Figure S4 shows the resulting models' fit and estimates for 2019 data, compared with the officially reported number of cases at each of the remaining provinces.

Disease (model)	Economic indicator (weight)	Touristic indicator (weight)	VFR indicator (weight)	Correlation with 2019 data	Overestimation
Malaria (simple)	Public limited companies (SA)	National travelers at hotels	Foreign residents by nationality	0.82	99.3%
Malaria (weighted)	No contribution (0)	No contribution (0)	Foreign residents by birthplace (1)	0.89	99.5%
Dengue (simple)	Public limited companies (SA)	National travelers at hotels	Foreign residents by birthplace	0.74	95.7%
Dengue (weighted)	Public limited companies (SA)	Overnight stays by national travelers at touristic apartments (0.1)	No contribution (0)	0.78	95.7%

Table S1: Summary of the models that most accurately approximated the 2015-2018 reported cases after excluding Madrid and Barcelona from the analysis. Each row shows the statistics that provided the best estimate of imported cases of each disease), the correlation with the actually reported data in 2019, and the proportion of overestimation as obtained from the linear models. For the models including weighted averages, the weight α_i of each indicator in included in parenthesis. If the weight of a given indicator is zero, this means that no contribution to the estimate is provided by the corresponding indicator.

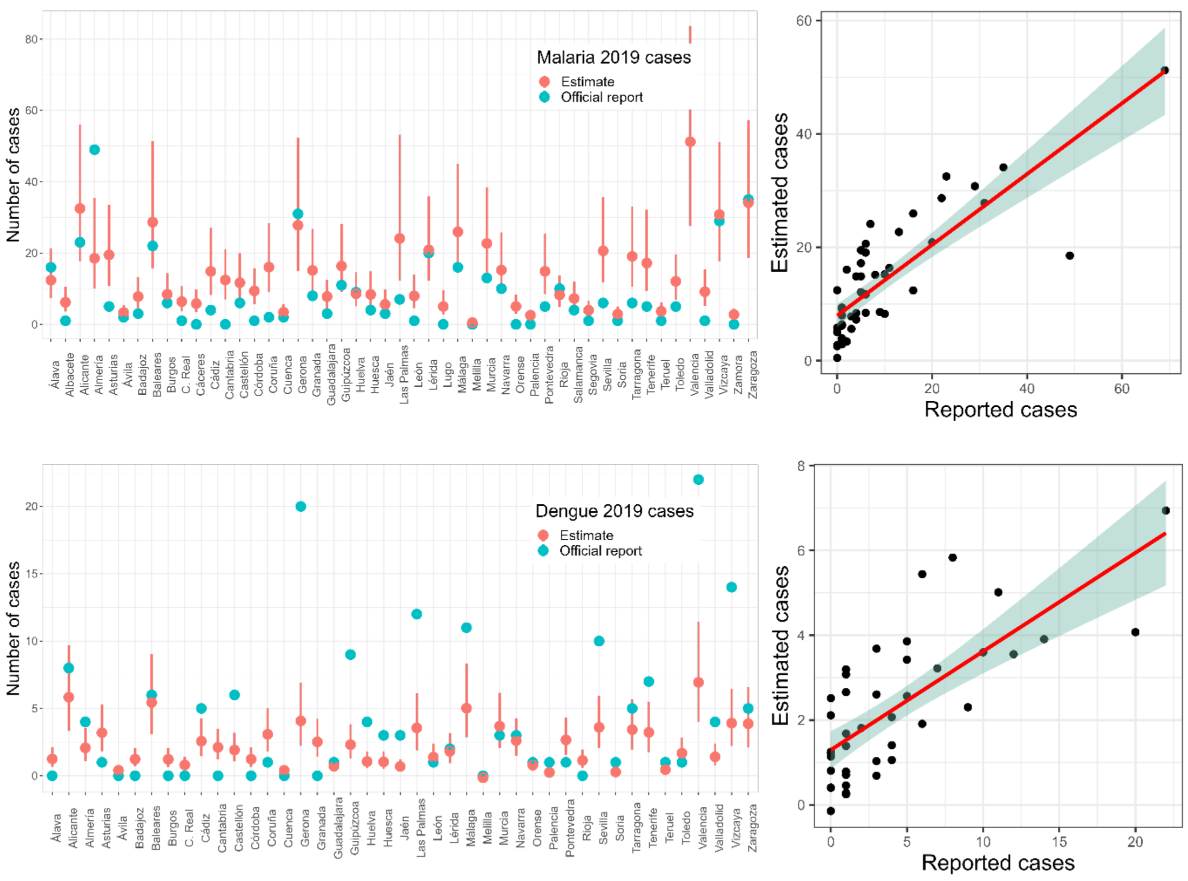

Figure S4. Summary of the best simple models for imported cases of malaria (top row) and dengue (bottom row), after excluding Madrid and Barcelona from the analysis. The left column shows the shows the models' predictions (in red), together with the actually reported cases (in blue) for 2019 for each province in Spain. The right column shows the fit between the models' estimates and the official records.

Human mobility models

Despite the relatively good score in terms of correlation with 2019 data found for the most accurate human mobility models (Table 3 in the main text), visual inspection of the models' fit showed that these results could be due to the dominance of some provinces from the dataset (Figure S5). After excluding these provinces from the analysis, the models' accuracy decreased significantly (Table S2). Average correlations with 2019 data in terms of models' parameters are provided in Figure S6 for general diagnostics.

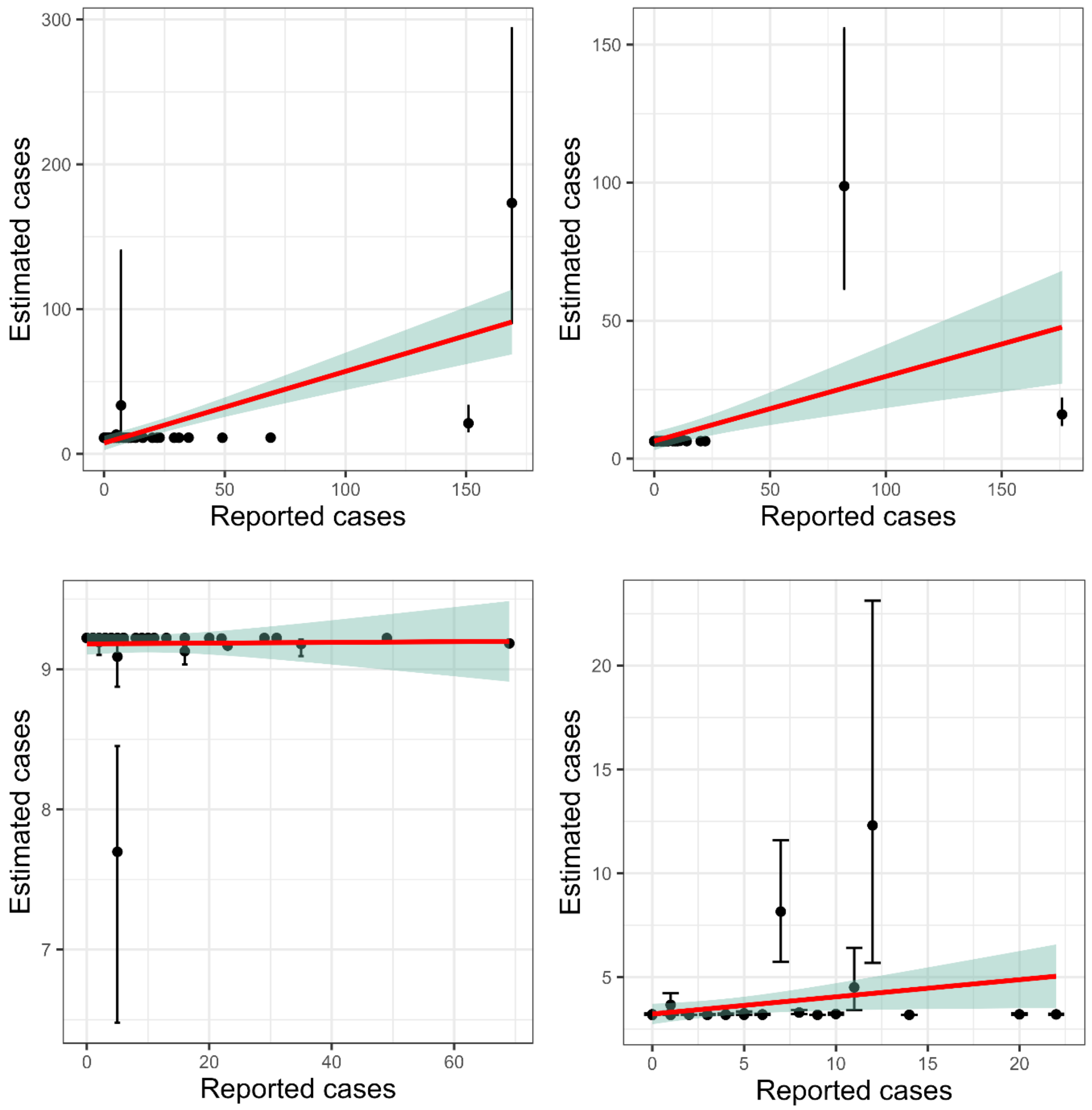

Figure S5. Models' fit for the human mobility models for malaria (left column) and dengue (right column). Top row shows the fit for the complete dataset of 52 provinces in Spain; bottom row shows the fit after removing the outliers from the analysis. Madrid, Barcelona and Las Palmas were removed from the malaria dataset, and Madrid and Barcelona were removed from the dengue dataset.

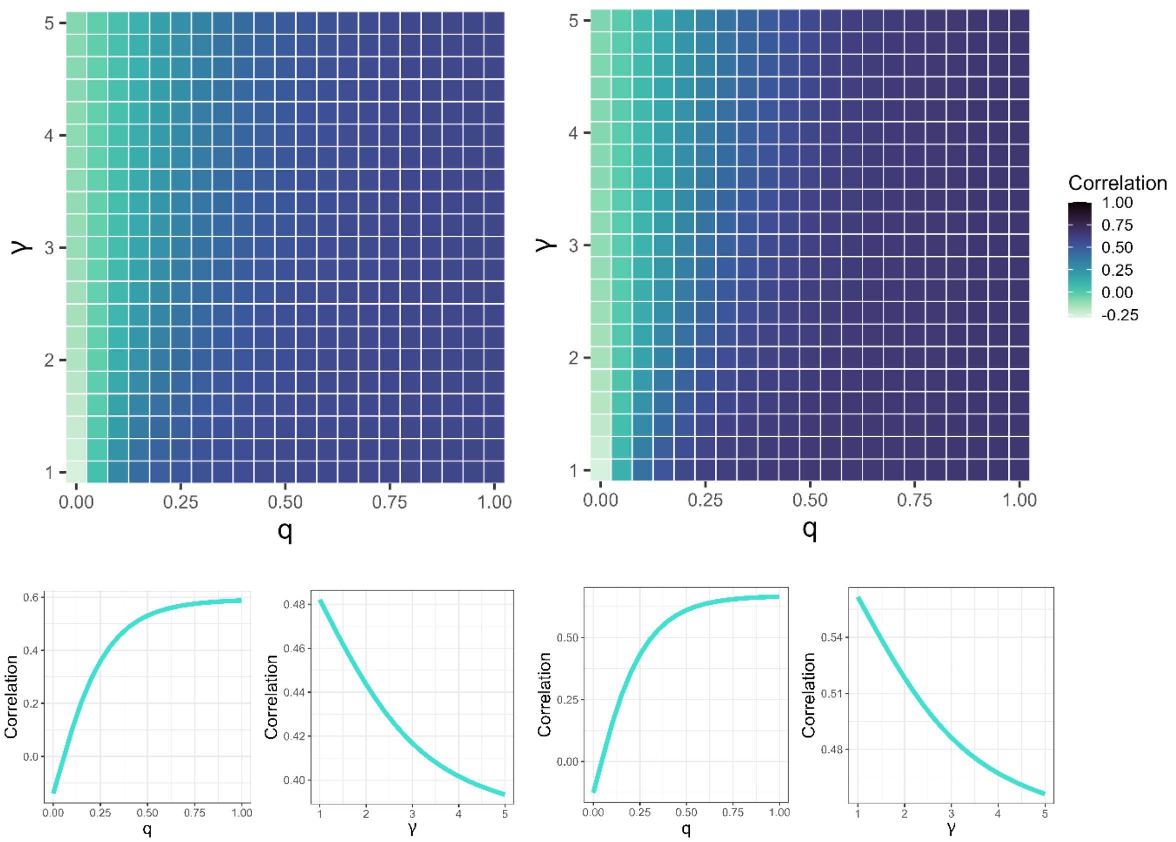

Figure S6. Accuracy of the human mobility model in terms of the models' parameters for malaria (left column) and dengue (right column). In each column, the top row shows the average correlation of the mobility models' estimates of imported cases with the 2019 official data in terms of the proportion of travelers that do not move after their arrival (q) and the exponent of the power law distribution (γ). Bottom row shows the average correlation of the models in terms of q and γ separately (left plot for q, right plot for γ).

Model	Proportion of cases that do not move after arrival (q)	Exponent of power law distribution (γ)	Correlation with 2019 data	Overestimation/ underestimation
Malaria	1	Any	0.003	97.7%
Dengue	1	Any	0.12	17%

Table S2: Summary of the human mobility models that most accurately approximated the 2019 reported cases after excluding outliers from the analysis. Each row shows the parameters of the model that provided the best estimate of imported cases of each disease, the correlation with the actually reported data in 2019, and the overestimation/underestimation of cases as obtained from the linear models. Madrid, Barcelona and Las Palmas were removed from the malaria dataset, and Madrid and Barcelona were removed from the dengue dataset.

Residual analysis

We analyze further the models' performance by inspecting their residuals, computed as the difference between the models' estimates and the reported cases. We performed a Shapiro-Wilk test, to check for normality of the residuals, and a Durbin-Watson test to check for autocorrelation. Table S3 shows the results of this analysis, and Figures S7-S9 show the distribution and QQ-plots of the models' residuals.

Model	Shapiro-Wilk test		Durbin-Watson test	
	Statistic	p-value	Statistic	p-value
Malaria	0.67	<0.001	2.03	0.543
Dengue	0.31	0.02	2.25	0.799
Dengue (excluding Barcelona)	0.94	<0.001	1.87	0.33

Table S3: Results of the residual analysis. Scores and p-values reported by the residuals of the weighted models in the Shapiro-Wilk and Durbin-Watson tests for normality and autocorrelation.

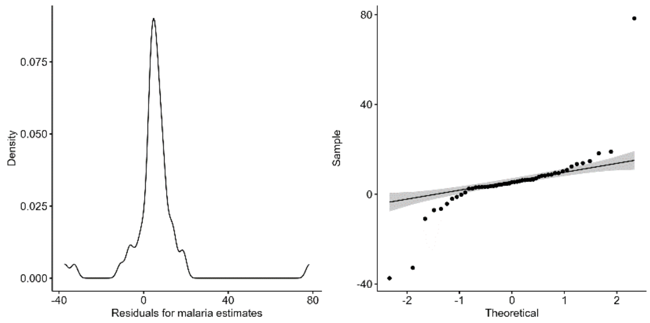

Figure S7. Residuals' density (left) and QQ-plot (right) for the malaria estimates. Plots obtained from the difference between the weighted model's estimate and the reported cases in 2019.

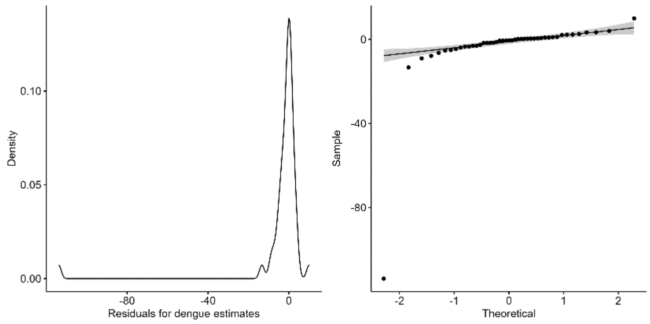

Figure S8. Residuals' density (left) and QQ-plot (right) for the dengue estimates. Plots obtained from the difference between the weighted model's estimate and the reported cases in 2019.

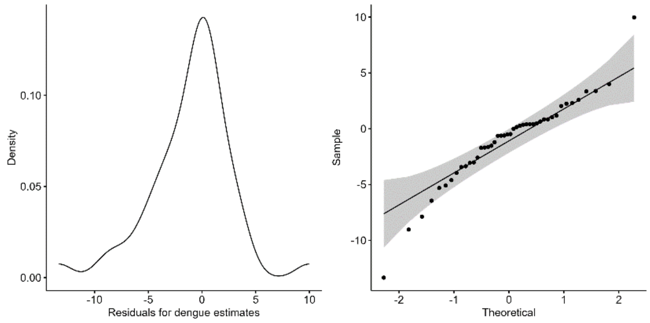

Figure S9. Residuals' density (left) and QQ-plot (right) for the dengue estimates, upon removing Barcelona from the analysis. Plots obtained from the difference between the weighted model's estimate and the reported cases in 2019.
